# Across two continents: The genomic basis of environmental adaptation in house mice (*Mus musculus domesticus*) from the Americas

**DOI:** 10.1371/journal.pgen.1011036

**Published:** 2024-07-05

**Authors:** Yocelyn T. Gutiérrez-Guerrero, Megan Phifer-Rixey, Michael W. Nachman

**Affiliations:** 1 Department of Integrative Biology and Museum of Vertebrate Zoology, University of California, Berkeley, California, United States of America; 2 Department of Biology, Drexel University, Philadelphia, Pennsylvania, United States of America; University of Virginia, UNITED STATES OF AMERICA

## Abstract

Replicated clines across environmental gradients can be strong evidence of adaptation. House mice (*Mus musculus domesticus*) were introduced to the Americas by European colonizers and are now widely distributed from Tierra del Fuego to Alaska. Multiple aspects of climate, such as temperature, vary predictably across latitude in the Americas. Past studies of North American populations across latitudinal gradients provided evidence of environmental adaptation in traits related to body size, metabolism, and behavior and identified candidate genes using selection scans. Here, we investigate genomic signals of environmental adaptation on a second continent, South America, and ask whether there is evidence of parallel adaptation across multiple latitudinal transects in the Americas. We first identified loci across the genome showing signatures of selection related to climatic variation in mice sampled across a latitudinal transect in South America, accounting for neutral population structure. Consistent with previous results, most candidate SNPs were in putatively regulatory regions. Genes that contained the most extreme outliers relate to traits such as body weight or size, metabolism, immunity, fat, eye function, and the cardiovascular system. We then compared these results with the results of analyses of published data from two transects in North America. While most candidate genes were unique to individual transects, we found significant overlap among candidate genes identified independently in the three transects. These genes are diverse, with functions relating to metabolism, immunity, cardiac function, and circadian rhythm, among others. We also found parallel shifts in allele frequency in candidate genes across latitudinal gradients. Finally, combining data from all three transects, we identified several genes associated with variation in body weight. Overall, our results provide strong evidence of shared responses to selection and identify genes that likely underlie recent environmental adaptation in house mice across North and South America.

## Author summary

Since their arrival with European colonizers, house mice have successfully spread throughout the Americas. There is strong evidence that populations in North America have adapted in that time, including parallel evolution of phenotypes across latitude (e.g., body size, behavior) as well as significant overlap of genes that show signals of selection. Here, we investigate the genetics of environmental adaptation in South America. We found that populations in South America are genetically distinct from populations in North America. We identified candidate genes for environmental adaptation with links to traits like body size, metabolism, immunity, eye function, thermoregulation and the cardiovascular system. We then bring together data from three transects across two continents to determine if environmental adaptation is predictable, with shared genetic responses. We found that most responses to selection do not involve changes in amino acid sequence and therefore are likely due to changes in gene regulation. We also found that while most candidate genes are unique to individual transects, there was more overlap than expected by chance. In addition, we observed parallel shifts in allele frequency among shared candidate genes, i.e., shifts in the same direction across different latitudinal gradients. These results suggest that there is a shared response to selection and identify a core set of candidate genes that likely contribute to environmental adaptation. Finally, we combine the data from all three transects to identify genes associated with variation in body weight. These findings highlight the value of studying wild populations of this important genetic model system.

## Introduction

Understanding the genetic details of how species adapt to new environments is a key goal of evolutionary biology. One approach to investigating the genetic basis of environmental adaptation is to look for covariation between allele frequencies and environmental variables [1,2]. Such clines can result from neutral processes, but statistical methods can be used to account for neutral population structure [e.g., 3,4], and this approach has been applied successfully to a wide range of organisms [5–9]. An extension of this approach is to compare patterns of genetic variation across multiple independent environmental gradients [e.g., 9–14]. For example, comparisons of *Drosophila melanogaster* populations in the northern and southern hemispheres have identified shared responses to selection [13,15,16]. When neutral population structure is accounted for, shared responses to selection and parallel clines, in particular, can provide strong evidence that particular genes and traits contribute to adaptation even when the specific mechanism is unknown. “Parallel evolution” is used to refer to a range of related patterns including similar shifts in phenotypes or alleles as well reuse of the same genes and/or pathways over independent gradients [e.g., 17–21]. For clarity, we refer to overlapping candidate genes as “shared” responses to selection, and we refer to shifts in allele frequencies or phenotypes in the same direction over an environmental gradient as “parallel” changes [e.g., 22–24].

Characterizing phenotypic variation in wild populations can be difficult, and biologically important phenotypes contributing to adaptation may go undetected. Even when there are known clines in phenotypes, many of the traits of interest may be polygenic and influenced by the environment. Detecting signatures of selection on complex traits and connecting those changes to phenotypes remains challenging [25–29]. However, signals of selection that are shared among clines can help identify genes and traits that contribute to adaptation. Moreover, because genome scans are agnostic with respect to phenotype, this kind of comparative approach can also point to previously unnoticed traits that may be important to adaptation.

House mice (*Mus musculus domesticus*) provide an opportunity to study the genomic basis of environmental adaptation using natural replicates. Native to Western Europe, house mice have spread opportunistically around the world in association with humans during the last five hundred years [30–34]. In this short time, they have successfully colonized both North and South America, from Tierra del Fuego (55°S) to Alaska (61°N), spanning an enormous range of habitats and climates. Previous studies have found that house mice exhibit clinal variation in body size, with size increasing with distance from the equator in South America and North America, consistent with Bergmann’s Rule [35–39]. House mice also show clines in ear length and tail length across North and South America, with length decreasing with increasing distance from the equator, consistent with Allen’s Rule [39]. These observations conform to well-known ecogeographic patterns in mammals and are thought to reflect thermoregulatory adaptations for animals living in cold or warm environments. These differences persist in a common laboratory environment for multiple generations, indicating that they have a genetic basis [36,38,39]. Genomic surveys have identified candidate genes using covariation between environmental variables and genetic variation in two clines across latitude in North America [36,38,40] in tandem with phenotype and gene expression data [36, 40]. Furthermore, comparison of the two latitudinal transects in North America identified significant overlap in signals of selection, including several genes related to heat sensing (e.g., *Trpm2*) and body weight (e.g., *Mc3r* and *Mtx3*), suggesting some shared response to selection [38].

Less is known about genetic variation in South American populations. Previous studies of altitudinal adaptation [41,42] and cytogenetics [43] provided evidence that house mice in South America derive from the same subspecies (*M*. *m*. *domesticus*) as mice in North America. However, sampling was limited, and it is not known whether there may be introgression from other subspecies. Patterns of genetic diversity and differentiation across the continent are also largely unknown as are the relationships to populations in North America. Importantly, some aspects of the environment, such as temperature, vary similarly across latitude in North and South America [44] providing an opportunity for an investigation of parallel rapid environmental adaptation across two continents.

Here, we explore genomic signatures of environmental adaptation in house mice from South America across a latitudinal transect from equatorial Brazil (~3° S) to southern Argentina (~ 55° S) using exome capture data of wild-caught individuals. We combined the data generated in this study with published data from eastern and western North America. We address five main questions. First, do house mice in South America derive from the same subspecies (*M*. *m*. *domesticus*) as house mice in North America? House mice are comprised of three major subspecies which diverged ~150,000–500,000 years ago and have distinct ranges: *M*. *m*. *musculus* is found in Eastern Europe and northern Asia, *M*. *m*. *domesticus* is found in Western Europe and the Mediterranean region, and *M*. *m*. *castaneus* is found throughout South and Southeastern Asia [45–48]. *M*. *m*. *domesticus* is the presumed source population for the Americas [30,49–51], although the subspecific origin of house mice across most of South America has never been explored. Second, are house mice in South America genetically distinct from populations in North America? If so, this would provide an opportunity to study the repeatability of evolution, including shared signals of selection and parallel evolution. Third, which genes show signatures of selection in house mice from South America? Fourth, to what extent are signatures of selection shared in comparisons among mice from three different latitudinal transects: South America (SA), eastern North America (ENA), and western North America (WNA)? Previous work showed that mice in eastern and western North America form two clades [38], providing an opportunity here to compare three phylogenetically independent transects. Finally, what genes underlie variation in body size and are they associated with signals of selection? We found that mice in South America are of *M*. *m*. *domesticus* origin and that they are more closely related to each other than to any populations in North America. We found signatures of selection across the genome among mice from South America across climatic gradients and we found significant overlap among candidate genes for all three transects, providing evidence of shared responses to selection. We also found that shifts in allele frequency at SNPs within overlapping candidate genes were typically in the same direction, suggesting that shared signals result from parallel evolution. Finally, a genome-wide association study (GWAS) identified eight genes associated with differences in body weight, all but one of which also showed signatures of selection.

## Results

### *Mus musculus domesticus* ancestry in the Americas

**Fig 1:**
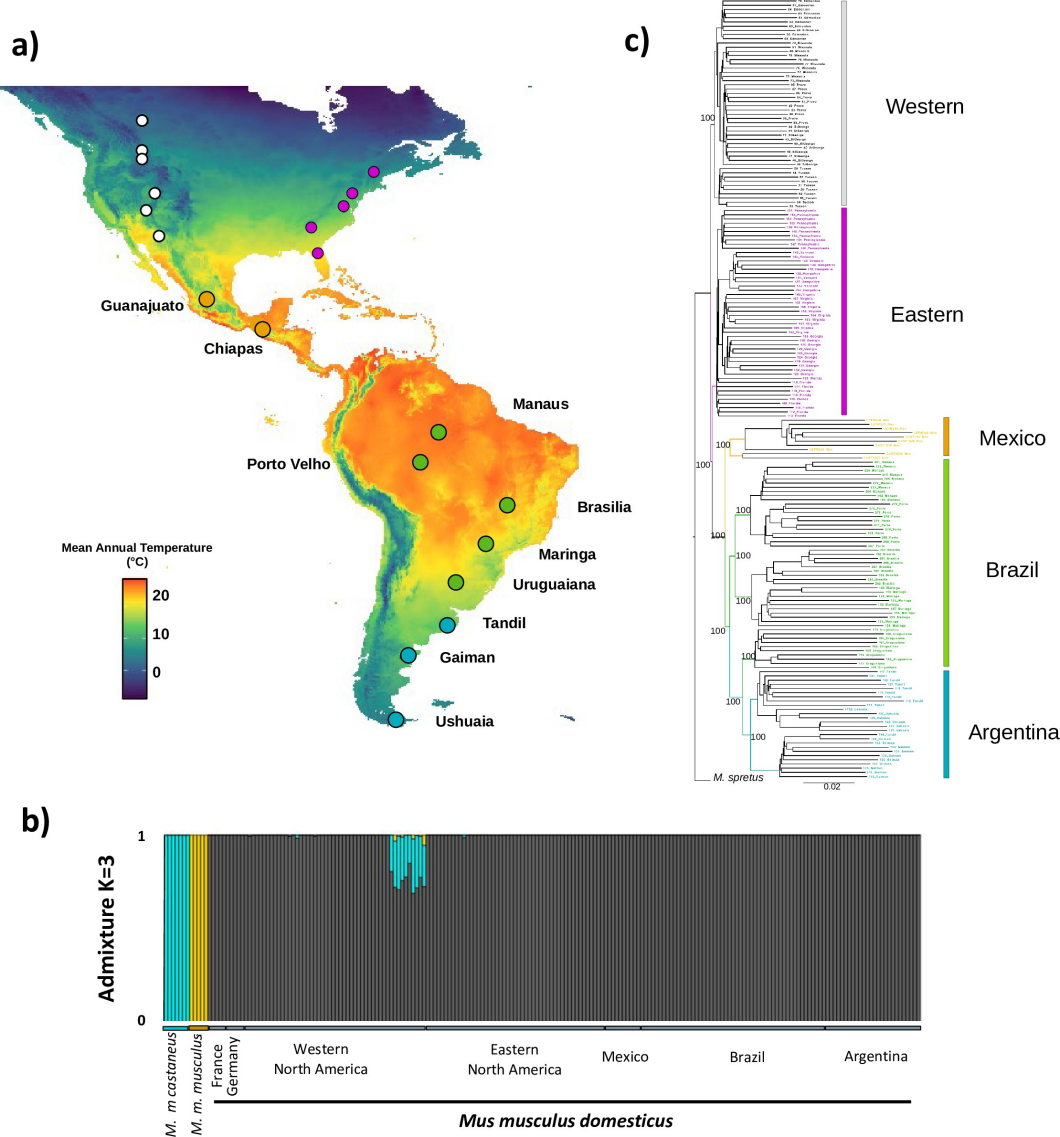
**a**) Map of mean annual temperature across the Americas (Map generated in R, using the WorldClim database information for Bio1- Mean Annual Temperature). Populations of wild house mice sampled across a latitudinal transect in Mexico and South America are shown with large circles. Populations included in previously published surveys in North America [36,38] are shown with small circles. **b)** Admixture plot including representatives from all three primary subspecies of house mouse as well as mice from sampled populations in the Americas. **c**) Phylogenetic reconstruction of *Mus musculus domesticus* populations across the Americas, with *M*. *spretus* as the outgroup.

We sequenced the complete exomes of 86 wild house mice sampled from 10 populations along a latitudinal transect from central Mexico to the southern tip of South America (Fig 1A and S1 Table). To analyze patterns of admixture, we combined these data with previously published data from populations in eastern (n = 50) and western (n = 50) North America [36,38] and published data from each of the three major *Mus musculus* subspecies [52] (S2 Table). Specifying K = 3 genetic clusters, we found that house mice in the sampled populations of the Americas are of *M*. *m*. *domesticus* origin, apart from one population in Tucson which is mostly of *M*. *m*. *domesticus* origin but also shows some limited admixture with *M*. *m*. *castaneu*s (Fig 1B), as previously reported [38]. These results provide strong evidence that house mice in Mexico and South America are *M*. *m*. *domesticus* with no evidence of significant introgression from the other subspecies.

### Phylogenetic relationships among transects in the Americas

We constructed a maximum likelihood phylogenetic tree using RAxML [53] with *M*. *spretus* as an outgroup (Fig 1C and S2 Table). For this analysis, we pruned the dataset to only include autosomal sites for which 80% of the individuals were covered, resulting in 895,333 sites. This analysis identified three major clades: populations from western North America, populations from eastern North America, and populations from Mexico and South America, each with 100% bootstrap support (Fig 1C). Within South America, mice formed two reciprocally monophyletic groups, each with 100% bootstrap support, largely corresponding to a northern clade (Manaus, Porto Velho, Brasilia, and Maringa) and a southern clade (Uruguaina, Tandil, Gaiman, and Ushuaia). In North America, mice formed two reciprocally monophyletic groups, each with 100% bootstrap support, corresponding to the eastern and western transects as previously reported [38]. Thus, these analyses indicate that mice within each transect are more closely related to each other than they are to mice in the other transects. This conclusion does not address whether selection has acted on new mutations, shared ancestral variation, or alleles introduced by rare long-distance migrants. House mice in the Americas derive from house mice in Western Europe within the last 500 years. Given the recency of this history, it is likely that selection acted mainly on ancestral shared variation or perhaps on alleles introduced through rare long-distance migration. However, the phylogenetic grouping of populations suggests that the response to selection occurred separately in each transect. For example, Fig 1C is inconsistent with the hypothesis that large-bodied mice far from the equator in the northern and southern hemispheres share a more recent common ancestor with each other than with the small-bodied mice closer to the equator within their transects.

### Population structure in the Americas

**Fig 2:**
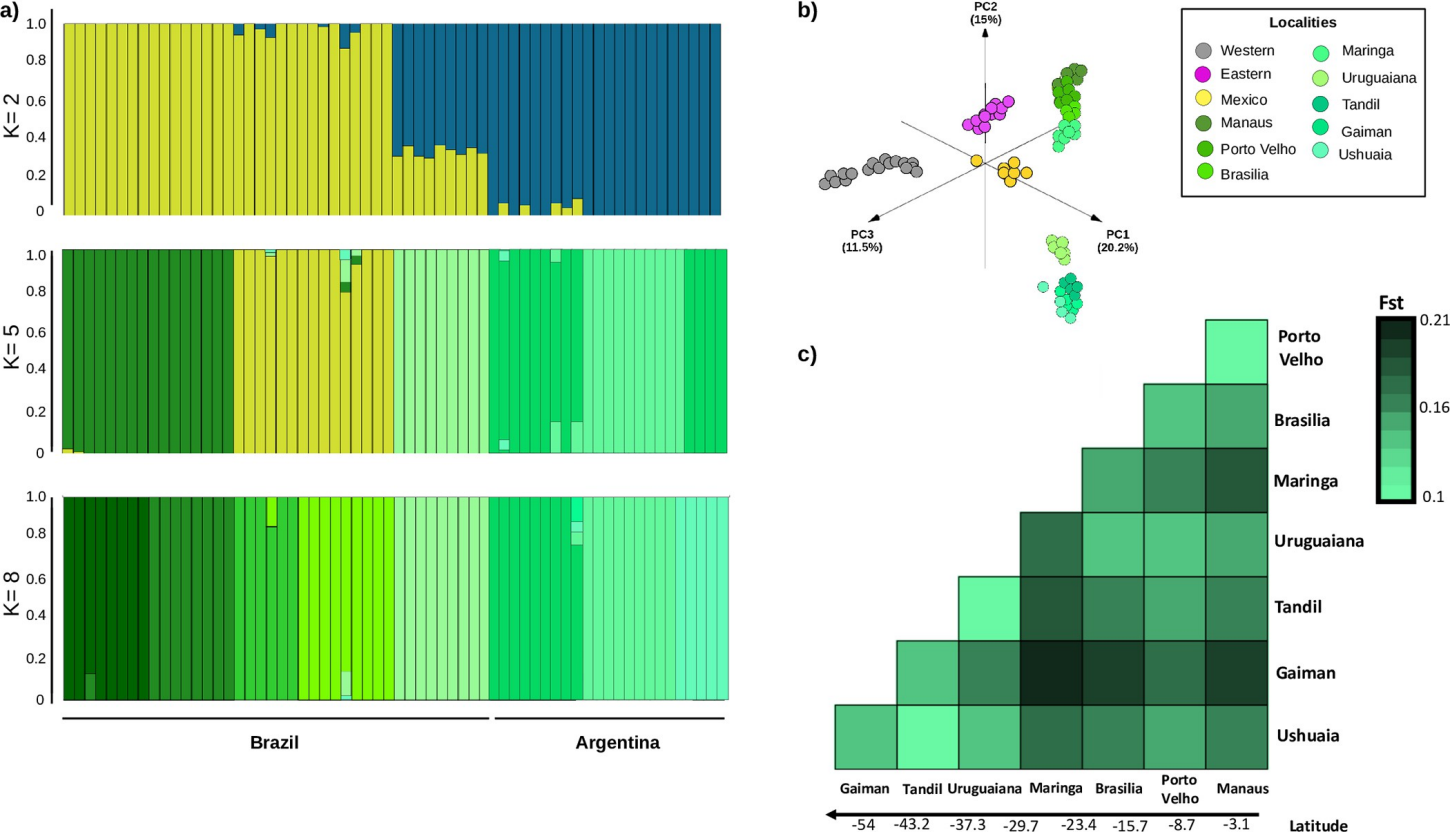
**a)** Admixture plot from South American populations evaluating K = 2, 5, and 8. **b)** Genetic PCA of *Mus musculus domesticus* populations across the Americas. **c)** Heatmap of pairwise genetic differentiation (*F*_*st*_) values between the eight populations in South America.

We first used NGSadmix to explore patterns of population structure within South America (Fig 2A), excluding Mexico because of limited sampling. The number of separate clusters that best fit the data was 5 (Evanno test). At K = 5 we observed clusters that corresponded to each sampled population or geographically close pairs of populations (Manaus and Porto Velho, Brasilia and Maringa, and Tandil and Ushuaia). At K = 8, each sampled population formed its own cluster. Consistent with the phylogenetic tree, principal component analysis including all populations in the Americas revealed genetic differentiation between North and South America, in which PC1 and PC2 largely separate populations by latitude (Figs 1C and 2B). When the first three principal components are plotted together, five major clusters are observed, corresponding to eastern North America, western North America, Mexico, northern South America, and southern South America.

The average *F*_*st*_ in pairwise comparisons among all eight South American populations (*F*_*st*_ = 0.154; Fig 2C) was significantly higher than the average pairwise *F*_*st*_ seen among the 10 North American populations (*F*_*st* Eastern_ = 0.069, Mann-Whitney U *p-value* = 0.00001, *z-score* = 4.52, and *F*_*st* Western_ = 0.079, Mann-Whitney U *p-value* = 0.00001, *z-score* = 4.6; S3 Table). We also detected a significant signature of isolation by distance among populations in SA (*Mantel statistic*: *R*^*2*^ = 0.2246, *p* = 0.0001; Fig 2C and S3 Table) in contrast to only modest evidence of isolation by distance in the WNA transect [38] and no evidence in the ENA transect [36]. The distance between sites is larger in the South American transect. However, even when limiting comparisons to similar geographic distances, there is a significant positive correlation between geographic and genetic distance in SA (y = 4x10^-5^x+ 0.091, *R*^*2*^ = 0.42), while there is no evidence of a correlation in ENA (y = -3x10^-6^ x +1.37, *R*^*2*^ = 0.0005). These differences suggest that barriers to gene flow (physical, political, or otherwise) may differ. While our sampling was not explicitly planned to test such hypotheses, rivers, topology, and regulated borders between countries within South America may result in more limited gene flow.

### Genomic signatures of environmental adaptation in house mice from South America

**Fig 3:**
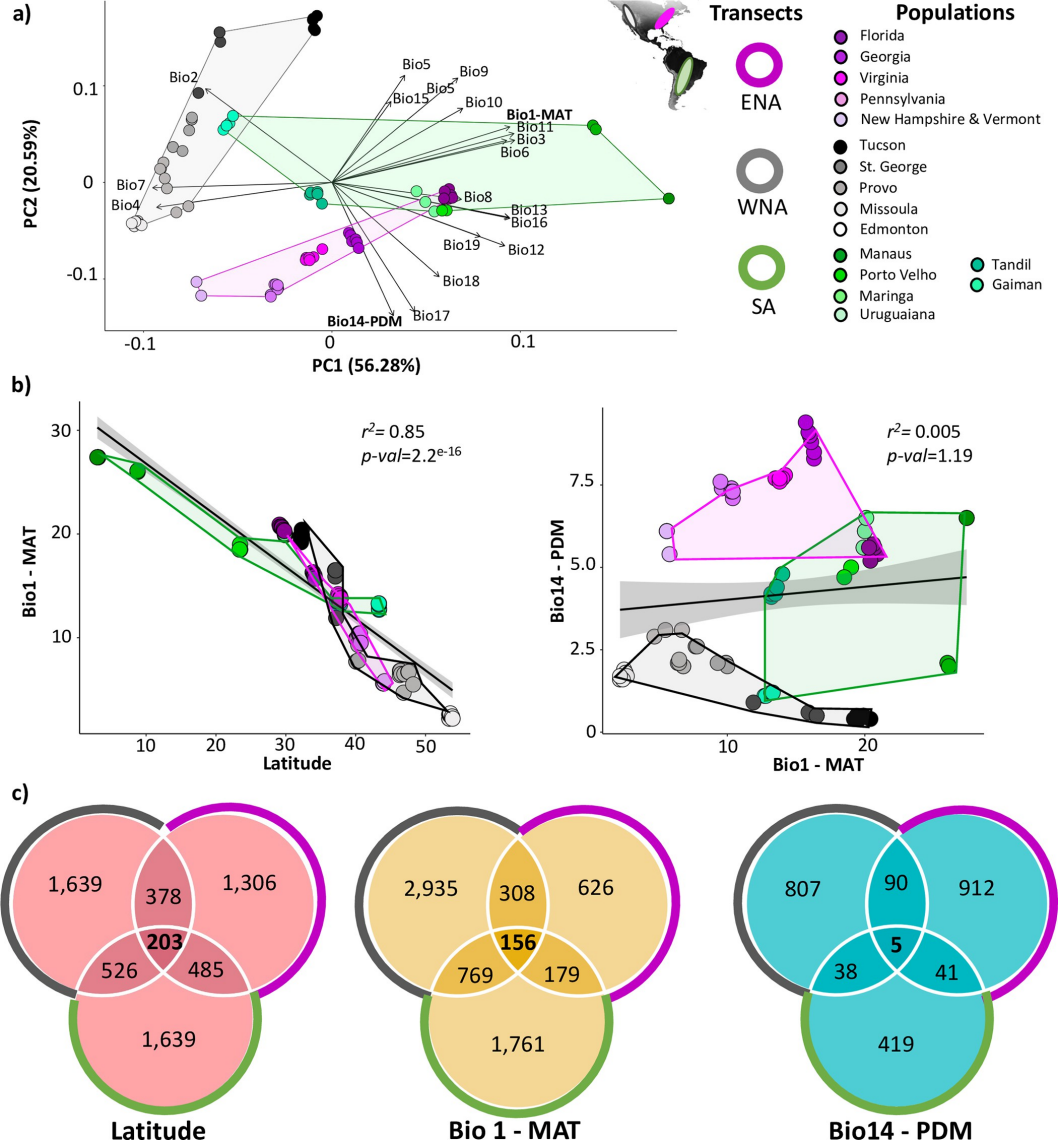
**a)** Climatic variation across the sampled localities in South America (SA), East (ENA) and West (WNA) of North America using PCA with 19 bioclimatic variables from the WorldClim database. The first component is mainly associated with variation relating to temperature (e.g., mean annual temperature, MAT), and the second principal component was mainly associated with precipitation of the driest month (PDM) and precipitation of the driest quarter. **b)** Latitude and Bio1- MAT are significantly correlated across the sampled localities, but there is no evidence of correlation between Bio14-PDM and Bio1-MAT across the sampled localities (ENA, WNA, SA). **c)** Venn diagrams illustrating the shared candidate genes across the three transects [South America (SA), Eastern of North America (ENA), and Western of North America (WNA)] for each variable. Map generated in R, using the WorldClim database information for Bio 1- Mean Annual Temperature (see https://github.com/YocelynG/HouseMouse_EnvAdapt).

We first explored variation in climatic variables across the sampled localities in South and North America. In a PCA analysis of the 19 bioclimatic variables from the WorldClim database, the first two principal components explained 71.53% of the total variance (Fig 3A and S4–S5 Tables). As expected for latitudinal sampling in this region, the first principal component was largely driven by mean annual temperature (MAT, Bio1) and other highly correlated temperature related variables. The second principal component was mainly associated with precipitation of the driest month (PDM, Bio14) and precipitation of the driest quarter (Bio17; Fig 3B). Since our sampling design was based on a latitudinal transect, we were most interested in exploring the effects of climatic variables associated with PC1, for which MAT showed a significant negative correlation with latitude (*R*^*2*^ = 0.85, *p-value* ≤ 2.2 x 10^−16^; Fig 3B). Mean annual temperature showed a very high degree of overlap among transects, varying from near 5°C to over 20°C in all three transects. In contrast, precipitation of the driest month showed variation in all three transects, but little overlap among transects (Fig 3B).

To identify candidate SNPs that show signals of environmental adaptation in South America, we used a latent factor mixed model (LFMM) that implements a Bayesian bootstrap algorithm [4]. This approach allows for the simultaneous identification of gene-environment correlations and estimation of confounding factors related to population structure (i.e., population history and isolation-by-distance), reducing the number of false positives [4]. Moreover, this approach is expected to perform comparatively well with respect to false positives when environmental factors that vary across a transect are correlated with population structure [54]. Closely related individuals were removed from the analysis (S1 Fig) as were individuals from Brasilia and Ushuaia due to insufficient sample size (N = 5), leaving 52 individuals from six populations (Manaus N = 8, Porto Velho N = 8, Maringa N = 9, Uruguaiana N = 9, Tandil N = 9, and Gaiman N = 9). Given those populations, we filtered SNPs retaining those with a minimum allele frequency of 5% and with sites called in at least 80% of all individuals (270,720 SNPs). We then conducted genomic scans for selection with LFMM at K = 3, first with latitude and then with MAT and PDM. We identified as outliers those sites with a |*z-score|* ≥ 2 and a *q-value ≤* 0.01 after False Discovery Rate (FDR) correction. Genes that contained outlier SNPs were considered candidate genes in all following analyses.

**Fig 4:**
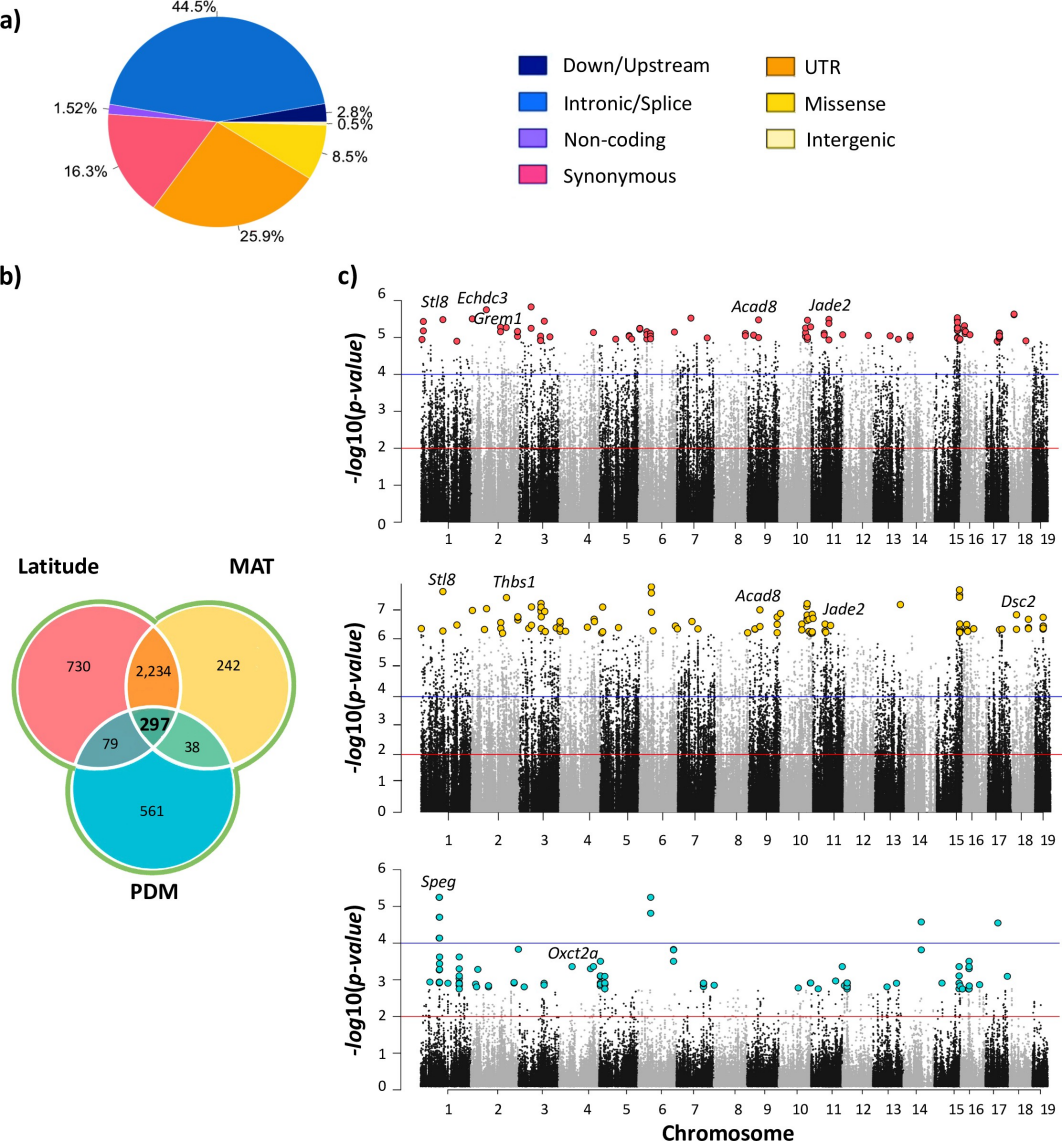
Signatures of selection among house mice from South America. **a)** The distribution of outlier SNPs across predicted variant effect categories. Proportions shown are averages across LAT, MAT, and PDM; full data are given in S7 Table. **b)** Venn diagram showing the number of unique and shared candidate genes in the South America transect for latitude, mean annual temperature (MAT), and precipitation of driest month (PDM). **c)** Manhattan plots showing the results of the population genomic scans for selection for the three environmental variables (red line indicates *q-value* = 0.05, and blue line indicates a *q*-*value* = 0.001). Highlighted are the first 100 hundred SNPs with the lowest *p-values* for each environmental variable.

We identified 9,600 outlier SNPs in >3,400 genes across the genome associated with variation in latitude and 7,481 outlier SNPs in >2,800 genes for MAT (Fig 4 and Tables 1 and S6). We identified far fewer candidate SNPs for PDM (1,007 SNPs in ~500 genes) which was expected given that the sampling scheme was not designed to explore variation in this variable (Figs 3, 4 and S6 Table). The vast majority of candidate SNPs were not amino-acid changing (Fig 4 and S7 Table) and most genes that contained a candidate SNP did not contain candidate SNPs that were amino-acid changing (S7 Table). We expect that candidate SNPs identified by the analyses are almost certainly not causative themselves, but rather are in linkage disequilibrium with causative variants. Nevertheless, most candidate genes did not have any non-synonymous candidate SNPs, suggesting that selection is acting mainly on regions that affect gene regulation. This finding is consistent with selection scans in the eastern and western transects of North America using latitude and mean annual temperature as variables [36,38].

**Table 1:** Genes annotated to top candidate SNPs in LFMM analyses of both latitude and mean annual temperature in the South American transect. Functional summarization is based primarily on MGI Mammalian Phenotype annotations and is not exhaustive.

MGI Symbol	Gene Name	Chr:Start:End	Candidate in other transect*	Gene Expression**	Adipose/ fat	Behavior	Blood/ glucose/ lipid homeostasis	Body Weight/ Body size	Cardiovascular system morphology/ function	Eye/retina morphology	Immunity	Renal/ urinary system
*A830018L16Rik*	RIKEN cDNA A830018L16 gene	1:11484329:12046125	ENA, WNA	Y	N	N	N	N	N	N	N	N
*St18*	suppression of tumorigenicity 18	1:6557455:6931164	WNA	N	Y	N	Y	Y	Y	Y	Y	N
*Grem1*	gremlin 1, DAN family BMP antagonist	2:113579020:113588993	ENA	N	N	N	Y	N	N	Y	N	Y
*Bcl2l11*	BCL2 like 11	2:127967958:128004467	ENA	Y	Y	N	Y	N	Y	N	Y	Y
*Echdc3*	enoyl Coenzyme A hydratase domain containing 3	2:6193276:6217805	--	Y	N	N	Y***	N	N	N	N	N
*Car13*	carbonic anhydrase 13	3:14706787:14728062	--	Y	N	N	Y	N	N	Y	N	N
*Tbl1xr1*	transducin (beta)-like 1X-linked receptor 1	3:22130816:22270758	--	Y	Y	N	Y	Y	Y	N	N	N
*Cald1*	caldesmon 1	6:34575433:34752404	--	Y	N	N	N	N	N	N	N	Y
*Agbl3*	ATP/GTP binding protein-like 3	6:34757367:34836394	--	N	N	N	N	N	N	N	N	N
*Cyren*	cell cycle regulator of NHEJ	6:34848706:34854995	--	Y	N	N	N	N	Y	N	Y	N
*Col28a1*	collagen, type XXVIII, alpha 1	6:7997808:8192617	WNA	N	N	N	N	N	N	N	N	N
*Arrdc4*	arrestin domain containing 4	7:68386742:68398986	--	Y	N	N	N	N	N	N	Y	Y
*Dscaml1*	DS cell adhesion molecule like 1	9:45338735:45665011	--	N	N	N	N	N	N	Y	N	N
*Sar1b*	secretion associated Ras related GTPase 1B	11:51654514:51682752	--	Y	N	N	Y	N	N	N	N	N
*Jade2*	jade family PHD finger 2	11:51704282:51748480	WNA	Y	N	N	Y	N	N	N	Y	Y
*Nlgn2*	neuroligin 2	11:69713949:69728610	--	N	N	Y	Y	N	N	N	N	N
*Plscr3*	phospholipid scramblase 3	11:69737202:69742884	WNA	Y	Y	N	Y	Y	N	N	N	N
*Galnt6*	polypeptide N-acetylgalactosaminyltransferase 6	15:100589694:100627257	--	N	N	N	Y***	N	N	N	N	N
*Kif21a*	kinesin family member 21A	15:90817479:90934151	WNA	Y	N	N	Y	N	N	Y	N	N
*Vwa5b2*	von Willebrand factor A domain containing 5B2	16:20408221:20424127	--	N	N	N	N	N	N	N	N	N
*Vps8*	VPS8 CORVET complex subunit	16:21241868:21463430	--	Y	N	N	N	N	N	N	N	N
*Ttc39c*	tetratricopeptide repeat domain 39C	18:12732953:12871920	ENA	Y	N	N	N	N	N	N	N	N
*Dsc2*	desmocollin 2	18:20163690:20192611	ENA, WNA	Y	N	N	N	N	Y	Y	N	N

*ENA: [36]; WNA: [38]

** Linked to differential expression, allele specific expression, and/or *cis*-QTL in comparisons among either wild mice from populations at different latitudes in the Americas [40] or lab strains derived from populations at different latitudes in the Americas [36,61,65]

***When no phenotypes were annotated, GO functional information was considered in summary categorization.

To explore the functional significance of candidates, we conducted gene ontology enrichment analyses for each set of candidate genes using Gowinda [55] and we used MouseMine and the Mouse Genome Database (via MGI) to identify phenotypes associated with the genes via mutants, knock-outs, and other genetic analyses [56–58]. Using Gowinda for gene enrichment analysis has the advantage of addressing potential biases introduced by variation in gene length [55]. Gowinda implements two analysis modes (*snp*, *gene*), both of which make simplifying assumptions regarding linkage between SNPs within a gene that can lead to over- and under- estimates of enrichment [55]. We applied both methods (S8 Table) and we used a third, modified approach in which we first pruned SNPs based on linkage disequilibrium (LD) and then ran the analysis in *snp* mode (see Materials and Methods and S8 Table). Results varied widely among these analyses. Analyses in *snp* mode yielded many enriched GO terms (~700–1400) for each candidate set, while analyses in *gene* mode yielded none. For brevity, we have focused on reporting results of the LD-pruned analysis which identified a modest set of enriched genes (but see S8 Table).

For latitude, we found significant enrichment (FDR ≤ 0.05; S8 Table) in a variety of biological processes including terms related to regulation and immunity (S8 Table). We also identified top candidate genes for latitude (genes annotated to the 100 SNPs with the lowest *p-values*) for which mutants are associated with phenotypes related to body weight and size, fat, metabolism (lipids, cholesterol, insulin, triglycerides, leptin, etc.), immunity, cardiac function, thermoregulation, limb and organ morphology, locomotion, and eye function/development, among others (Tables 1 and S6, S8). Functional results for MAT were similar to those for latitude (Tables 1 and S8). For PDM, we detected candidate genes with diverse mutant phenotypes including those related to immunity, muscle function, and kidney function/morphology (S8 Table). There was enrichment of biological process GO terms including those related to regulation of hormone levels, regulation of metabolic processes, metal ion transport, and ion homeostasis, among others (S8 Table).

As expected, most of the candidate SNPS and genes identified using latitude and MAT were shared (>6,541 shared SNPs from 2,545 genes and 2,575 shared genes including those for which there was not exact overlap at the SNP level), whereas candidates identified using PDM were largely unique (Fig 4B and 4C). Twenty-three genes were annotated to the 100 SNPs with lowest corrected p-values for both latitude and MAT (Table 1). These genes are linked to many mammalian phenotypes (MGI [56–58]; Tables 1 and S8) including abnormal blood homeostasis (*Bcl2l11*, *Grem1*, *Jade2*, *Kif21a*, *Nlgn2*, *Plscr3*, *Sar1b*, *St18*, *Tbl1xr1*), lipid and fatty acid metabolism, body fat, and related phenotypes (*Bcl2l1*, *Plscr3*, *Sar1b*, *St18*, *Tblxr1*), abnormal glucose homeostasis *(Car13*, *Jade2*, *Kif21a*, *Plscr3*, *Tbl1xr1)*, immunity (*Arrdc4*, *Bcl2l11*, *Cyren*, *Jade2*, *St18*), abnormal eye/retina morphology (*Car13*,*Dsc2*, *Dscaml1*, *Kif21*, *St18)*, abnormal renal/urinary system (*Arrdc4*, *Bcl2l11*, *Cald1*, *Grem1*, *Jade2*), abnormal cardiovascular system morphology (*Cyren*, *Dsc2*, *St18*, *Tbl1xr1*), abnormal limb and digit morphology (*Grem1*), and behavior (*Nlgn2*; Table 1). Four of these 23 genes included top candidate SNPs classified as missense modifications (*Agbl3*, *Cald1*, *Vwa5b2*, *Vps8)*. In humans, *Plscr3* and *Sar1b* are linked to obesity and lipid metabolic disease, respectively, *Kif21a* is linked to ocular disease, and *Nlgn2* is linked to schizophrenia (MGI [56–58]).

### Shared response to selection across transects in the Americas

The three transects were planned to capture responses to climatic variables that covary with latitude, allowing us to investigate the extent to which responses to selection are shared. As expected, all three transects have similar ranges in MAT, from near 5°C to over 20°C, although the shift is more gradual in South America, covering ~50 degrees of latitude compared to ~20 degrees of latitude in the other transects (Figs 1A and 3B; S5 Table). The transects were not planned to cover clines in PDM and there was little overlap in the range of values among transects in PDM (Fig 3A and 3B), but we also considered this variable given its inclusion in the analysis of South American populations and since it reflects climatic variation that is largely orthogonal to MAT (Fig 3A and 3B)

To ensure standardization, we repeated genome scans using published exome-capture data for transects in eastern North America (50 individuals in five populations) [36] and western North America (50 individuals in five populations; Fig 1A and S1 Table) [38] using exactly the same approach to calling SNPs and genotypes as for the South American transect. After filtering, there were 281,362 SNPs in the transect from eastern North America and 342,108 SNPs in the transect from western North America (minimum allele frequency of 5%, with sites called in at least 80% of all individuals). For each transect and variable (latitude, MAT, and PDM), we identified candidate SNPs applying a *q-value* cut-off ≤ 0.05 and |*z-score| ≥* 2 (S9 Table). To identify shared responses to selection among transects, we compared the candidate genes identified using each environmental variable in pairwise comparisons between transects (S10 Table). We performed permutation tests using 100,000 replicates with replacement to evaluate whether the overlap in shared genes in pairwise comparisons was significantly greater than expected by chance (S11 Table).

In each of these three pairwise comparisons, the number of shared candidate genes was significantly greater than expected by chance in all analyses of latitude and MAT as evaluated via permutation test (*z-score* ≥ 3, *p-value ≤* 0.005 for each of the six comparisons; Fig 3C and S10, S11 Tables). Overlap among candidates identified using PDM was significantly more than expected by chance in only one comparison (WNA-ENA, *z-score* = 2.65, *p-value =* 0.007; Fig 3C and S10, S11 Tables). The proportion of shared genes was also much higher for latitude ($\bar{x}$ = 16.50%) and MAT ($\bar{x}$ = 16.22%) than for PDM ($\bar{x}$ = 6.95%; Fig 3C). When considering the overlap among all three transects, the pattern was even more pronounced. The proportion of genes shared among all three transects for latitude ($\bar{x}$ = 7.30%) and MAT ($\bar{x}$ = 7.16%) was more than seven times greater than for PDM ($\bar{x}$ = 0.93%; S10 Table). These observations are consistent with the overlap in climatic variables relating to temperature but not precipitation among transects (Fig 3A and 3B). Nevertheless, while there was more overlap among transects than expected by chance for latitude and MAT, most signals of selection were specific to individual transects (Fig 3C).

We explored the function of candidate genes shared among the three transects for each environmental variable. For latitude, 203 genes were shared in the three transects with diverse annotated phenotypes (MGI [56–58]) including those related to metabolism (insulin, glucose, leptin, lipids, cholesterol, etc.), body size/fat, immunity, reproduction, eye development/function, behavior, thermoregulation, and cardiovascular function/development (Fig 3C and S10, S12 Tables). Links to cardiovascular function suggest possible impacts on thermoregulation (for example, an efficient mechanism to avoid blood vessel constriction when temperature drops) [59,60]. For MAT, we found that 156 genes were shared, with a similar range of functions as for latitude (Fig 3C and S10, S12 Tables). Only five candidate genes were shared among transects for PDM (*Cgnl1*, *Col27a1*, *Myo15*, *Robo1*, *Sri*; Fig 3C).

In total, 90 genes were identified for both latitude and MAT in all three transects (S12 Table). Of these 90 genes, 24 were top candidates (annotated to the 200 SNPs with lowest *p-values*) in at least one transect (Table 2). Many of these have mutant phenotypes related to abnormal behavior (12), abnormal homeostasis (11) and related child terms, abnormal immune system morphology (6), and abnormal body size (6). Over half have GO annotations related to metabolic processes (14). Notable among these are genes like *Mc3r* which was identified as a top candidate in both North American transects and is functionally linked to body size and metabolism (Table 2). *Rorb* was a top candidate in two transects and linked to differences in gene expression in strains derived from the Americas [61]. *Rorb* is a nuclear receptor that functions in photoreceptors. Normal development of rod and cone photoreceptor cells depends on *Rorb* [62] and it is known to be involved in the regulation of circadian rhythm [63,64]. *Akap9* which was a top candidate in the South American transect, is linked to differences in gene expression in strains derived from the Americas [36,61,65], and has annotated phenotypes relating to fat, immunity, and metabolism.

**Table 2:** Candidate genes shared among all three transects for both latitude and mean annual temperature that were a top candidate for at least one of the variables in at least one transect. Functional summarization is based primarily on MGI Mammalian Phenotype and GO Biological Process annotations and is not exhaustive.

MGI Symbol	Chr:Start:End	Gene Name	Top Candidate*	Linked to changes in gene expression**	Parallel shifts***	Functional Summary
*A830018L16Rik*	1:11484329:12046125	RIKEN cDNA A830018L16 gene	SA	Y	Y	NA
*Ahctf1*	1:179572459:179631245	AT hook containing transcription factor 1	ENA	Y	N	Regulation of cell cycle, chromatin, nuclear pore, cell mass
*Mgat4a*	1:37478421:37580097	mannoside acetylglucosaminyltransferase 4, isoenzyme A	ENA	Y	Y	Abnormal blood homeostasis, glucose/insulin/fatty acids, weight gain
*Mc3r*	2:172090412:172093034	melanocortin 3 receptor	ENA, WNA	N	N	Body size, fat, leptin, insulin, food intake
*Myo3a*	2:22232314:22508264	myosin IIIA	WNA	N	Y	Hyperactivity, hearing
*Hc*	2:34873343:34951450	hemolytic complement	WNA	Y	Y	Grip strength, glucose, immunity
*Ndst3*	3:123319815:123484502	N-deacetylase/N-sulfotransferase (heparan glucosaminyl) 3	SA	Y	N	Cholesterol, immunity
*Pde4dip*	3:97597144:97796023	phosphodiesterase 4D interacting protein (myomegalin)	WNA	Y	Y	Hyperactivity, hemoglobin, bone mineral, retina/cornea
*Wdfy3*	5:101980822:102217787	WD repeat and FYVE domain containing 3	SA	Y	Y	Craniofacial/liver/spleen/brain/blood vessel morphology
*Fry*	5:150042110:150421218	FRY microtubule binding protein	WNA	Y	Y	Mitosis
*Dpp6*	5:27022355:27932498	dipeptidylpeptidase 6	WNA	Y	Y	Abnormal nervous system morphology
*Akap9*	5:3977410:4130204	A kinase anchor protein 9	SA	Y	Y	Homeostasis, cholesterol, calcium, spermatogenesis, tooth morphology, body weight, immunity, fat
*Mical3*	6:120908668:121107959	microtubule associated monooxygenase, calponin and LIM domain containing 3	ENA	Y	Y	Immunity, activity
*Tspan11*	6:127864585:127930940	tetraspanin 11	ENA	Y	Y	Coat/hair, vocalization
*Met*	6:17463799:17573979	met proto-oncogene	WNA, SA	Y	N	Metabolism, cancer, pulmonary function/morphology, liver, brain
*Cdh8*	8:99751103:100143103	cadherin 8	WNA	Y	Y	Thermal nociception, tail movement, abnormal nervous system physiology
*Mlh1*	9:111057296:111100854	mutL homolog 1	ENA, SA	Y	Y	Fertility, immunity
*Wdr72*	9:74017608:74190485	WD repeat domain 72	ENA	Y	Y	Body weight, enamel/tooth
*Fat2*	11:55141435:55227390	FAT atypical cadherin 2	ENA	N	Y	Cell-cell adhesion
*Slc35b1*	11:95275696:95282602	solute carrier family 35, member B1	WNA	N	Y	Energy metabolism
*Tspan13*	12:36064554:36092477	tetraspanin 13	WNA	Y	Y	Bone density/structure, body fat, glucose
*Hivep1*	13:42205304:42338504	human immunodeficiency virus type I enhancer binding protein 1	SA	Y	N	Heart morphology
*Epha3*	16:63363897:63684538	Eph receptor A3	WNA	Y	Y	Heart, spleen, body size
*Rorb*	19:18907969:19088560	RAR-related orphan receptor beta	ENA, WNA	Y	Y	Eye, body size, coordination, regulation of circadian rhythm

*ENA: [36]; WNA: [38]

** Linked to differential expression, allele specific expression, and/or *cis*-QTL in comparisons among either wild mice from populations at different latitudes in the Americas [40] or lab strains derived from populations at different latitudes in the Americas [36,61,65].

***Genes marked with “Y” have shared SNPs that show the same direction of allele frequency change across all three transects (S13 Table).

In addition to identifying shared responses to selection at the gene level, we were also interested in determining if there were parallel shifts in SNP allele frequencies. There were no candidate SNPs that overlapped among all three transects. This may be due in part to the modest depth of sequencing and the fact that the SNPs with sufficient data to estimate allele frequencies varied in each transect. In addition, candidate SNPs are not expected to be causative, but rather in linkage with causative variants. Given the age of these populations, though, haplotypes are likely largely shared [40]. Therefore, to investigate parallelism, we first identified all candidate genes that were shared among the three transects. Next, we identified the shared SNPs within those candidate genes with sufficient data from all of the populations at the ends of each transect to estimate allele frequencies. We then asked whether the direction of the allele frequency shift across the climatic gradient was the same among the three transects for each of those SNPs. In other words, we counted the number of genes for which there was a parallel shift in allele frequency from populations at higher latitudes to those at lower (i.e. Edmonton vs Tucson; New Hampshire–Vermont vs Florida; and Gaiman vs. Manaus; S13 Table). We found a total of 101 genes (out of 173 shared candidates with sufficient data) for latitude, 116 genes (out of 150) for MAT, and five genes (out of five) for PDM for which the direction of allele frequency shifts was shared in all three transects. The number of genes with parallel changes in allele frequencies across all three transects was significantly more than expected by chance for all variables (latitude, MAT, and PDM) using both a χ^2^ and permutation test (S13 Table).

### GWAS for body weight and body mass

**Fig 5:**
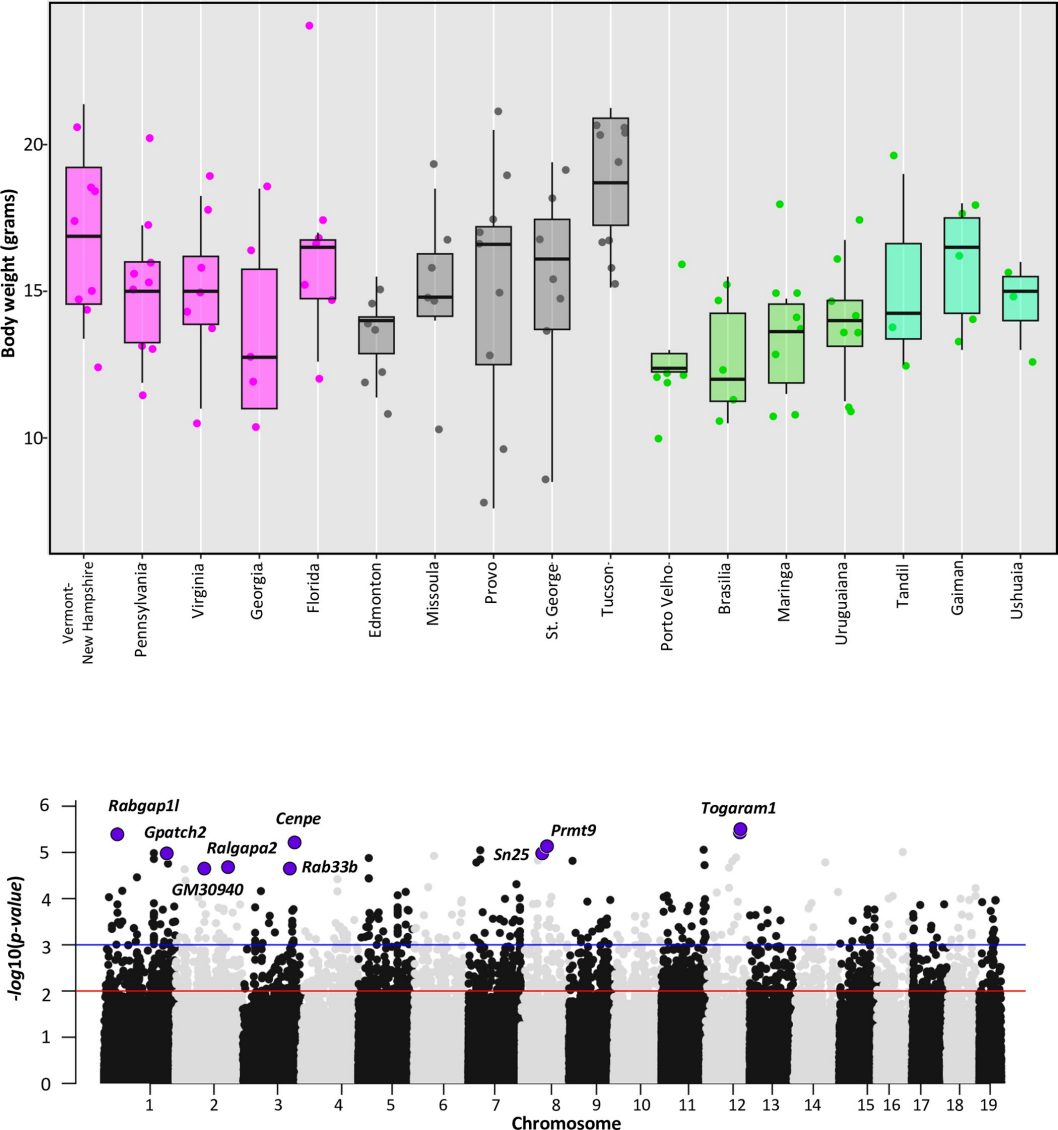
Body weight and genes associated with body weight in house mice from North and South America. **a)** Boxplot shows the distribution of adult body weights for each population from Eastern North America (magenta), Western North America (grey), and South America (green). Pregnant females have been excluded. **b)** Manhattan plot showing the genome-wide association results using exome and body weight data. Significant candidate SNPs (FDR ≤ 0.05) associated with body weight are highlighted in purple with the gene annotated for the SNP (red line indicates *p-value* = 0.01, and blue line indicates a *p*-*value* = 0.001). Description of the genes is in Table 3 and detailed GEMMA results are given in S14 Table.

Consistent with Bergmann’s rule, body size in house mice varies clinally in both North America and South America, with larger animals farther from the equator [36–40] (Fig 5). Body weights of wild-caught mice are positively correlated with degrees from the equator in ENA and SA but not in WNA, as previously reported [37] (Fig 5). However, data from field collections can be noisy, reflecting differences in age and environmental conditions. Age matched lab-born descendants of mice from Edmonton are significantly larger than lab-born descendants of mice from Tucson [38], consistent with patterns seen in ENA [36]. To identify genes contributing to variation in body weight, we performed an association study using the exome data from 114 adult mice from South America (n = 38), ENA (n = 36), and WNA (n = 40). We excluded juvenile animals, pregnant or lactating females, and mice from Manaus which were housed in a laboratory setting before weighing. After filtering (MAF of 5% or greater and less than 10% missing data), 163,121 SNPs remained. We repeated the analysis using body mass index (BMI).

We carried out the analysis via GEMMA [66] using sex as a covariate, controlling for relatedness among individuals, and correcting for multiple testing (FDR ≤ 5%). We did not find SNPs significantly associated with variation in BMI. We did find 10 SNPs in eight genes and one gene model (*Cenpe*, *Gm30940*, *Gpatch2*, *Rab33b*, *Rabgap1l*, *Ralgapa2*, *Prmt9*, *Snx25*, *Togaram1*) significantly associated with variation in body weight (FDR ≤ 0.05, Tables 3 and S14). None of these genes were identified in a similar GWAS limited to the western North American transect [38], but all but one was identified as a candidate gene in scans for selection using LFMM (Tables 3 and S14). Overlap between the GWAS and LFMM selection scan candidates is significantly more than expected by chance using a permutation test (*z-score* = 4.64, *p-values* < 0.005). Mutants in these genes relate to varied phenotypes, including aspects of metabolism, immunity, and morphology (MGI [56–58]). For example, mutations in *Cenpe* are associated with phenotypes related to lipase and glucose levels and *Cenpe* was differentially expressed in fat tissue collected from lab raised mice from New York and Florida [36]. Immunity phenotypes are annotated to *Gpatch2*, *Rab33b*, and *Prmt9*. Mutations in *Rab33b* also relate to bone morphology, body weight and cardiac function and *Prmt9* is also annotated to phenotypes relating to metabolism and grip strength (Table 3).

**Table 3:** Genes annotated to candidate SNPs identified via GEMMA analysis of body weight across the Americas. Functional summarization is based primarily on MGI Mammalian Phenotype annotations and is not exhaustive. For additional detail on GEMMA results, see S14 Table.

MGI Symbol	Gene Name	Location	*p*-value	MGI Phenotype	Candidate in other transect
*Rabgap11*	RAB GTPase activating protein 1-like	1:160236459	1.27e^-6^	- -	ENA, SA
*Gpatch2*	G patch domain containing 2	1:187341647	8.88e^-6^	Immunity	- -
*Gm30940*	Gene model	2: 73563937	8.73e^-6^	- -	- -
*Ralgapa2*	Ral GTPase activating protein, alpha subunit 2	2: 146447211	5.83e^-6^	Neoplasm, renal/urinary system; glucose metabolism in adipocytes*	ENA WNA, SA
*Rab33b*	Member RAS oncogene family	3:51483947	8.39e^-6^	Bone morphology/function, body weight, increased aspartate transaminase level (associated with cardiac events/hepatitis)	WNA
*Cenpe*	Centromere E protein	3:135242350	5.12e^-6^	Homeostasis/metabolism, chromosomal stability, liver regeneration	WNA
*Snx25*	Sortin nexin 25	8: 46593877	5.33e^-6^	Activity, cardiac function; circadian pace-making**	ENA, WNA, SA
*Prmt9*	Protein arginine methyltransferase 9	8: 77581031	5.83e^-6^	Behavioral/neurological, hearing/vestibular/ear/hematopoietic system, immune system	ENA, SA
*Togaram1*	TOG array regulator of axonemal microtubules 1	12: 65057994 12:65062969	1.41e^-6^ 5.66e^-6^	Facial cleft; Abnormal limb bud morphology; Polydactyly	ENA, SA

*[67]

**[68]

## Discussion

### Genetic variation in the Americas

We now have a comprehensive view of genomic variation in wild house mice across the Americas [36,38] (Fig 1A). Our data strongly support that the populations surveyed in Mexico and South America derive from *M*. *m*. *domesticus*, the subspecies found in Western Europe, consistent with known European colonization of the Americas [30,34]. Overall, contributions from other subspecies appear to be restricted to southwestern North America, with signals of admixture from *M*. *m*. *castaneus* in Tucson, AZ [38]. Previous studies of retroviral resistance showed that mice in the Lake Casitas region of southern California showed significant introgression from *M*. *m*. *castaneus* [69] which may be linked to immigration from China [70]. Gene flow between southern California and Tucson would have been facilitated by the launch of a railroad line linking the areas in 1880 [71]. Additional sampling in California and the Southwest would help determine the extent to which introgression from *M*. *m*. *castaneus* has contributed to genetic variation in Western North America.

We found clear patterns of genetic differentiation among populations and among the three transects. Importantly, patterns of genetic variation across the continents suggest that the three sampled transects are genetically distinct. Geographic distance was not strongly predictive of levels of differentiation among populations within transects in North America [36,38]. Idiosyncratic patterns may reflect complex but porous barriers to long distance gene flow due to human mediated transport (contemporary and/or historic). In contrast, geographic distance was a good predictor of genetic distance in South America, which may reflect more effective barriers to gene flow (e.g., elevation, regional climates, water ways, political borders) within the transect. Interestingly, Brazil and Argentina formed a clade sister to the Mexican clade. The clustering of Mexico with South American populations is somewhat surprising based on the significant physical barriers between Mexico and populations in Brazil and Argentina and the comparative proximity to southern populations in the WNA transect. While speculative, the existence of the South/Central American clade suggests the hypothesis that historic patterns of European colonization (e.g., colonization predominately by Spain and Portugal in Central and South America vs. England and France in North America) have influenced population structure in house mice in the Americas. These data complement genomic data from European populations, opening the door to a higher resolution understanding of the population genetics of an invasive species [34].

### Environmental adaptation in South America

In South America as in North America, we identified signals of selection associated with variation in latitude and mean annual temperature, with many of the candidate genes related to metabolism, fat, and body size, consistent with the observed variation in body size among mice from different latitudes [37] (Fig 5). Top candidate genes were also linked to phenotypes related to immunity and cardiac, eye, and renal/urinary systems. We also considered PDM, identifying candidate genes with diverse functions including those related to immunity, muscle function, kidney function/morphology, and cholesterol.

Nearly half of top candidate genes associated with variation in latitude and mean annual temperature in South America were also identified as candidates in previous studies of environmental adaptation in mice in the Americas and over half of them have been linked to differences in gene expression either in lab strains derived from these populations in the Americas or from wild populations in the ENA transect. Ten of these genes have mutant/knockout phenotypes related to blood/glucose/lipid homeostasis. These genes represent excellent candidates for additional investigation of the genetic basis of environmental adaptation in South America.

### The challenge of investigating adaptive complex traits in wild populations

Genome-wide scans can be powerful tools for detecting selection in wild populations. Agnostic to phenotype, they allow us to capture signatures of selection even when phenotypic variation may go unnoticed or be difficult to characterize. However, one major challenge of genome scans is that it can be difficult to connect candidate SNPs and genes to effects on traits that affect fitness. The depth of functional work in house mice as a genetic model can be useful, helping to link candidate genes to function via mutants, knock-outs, and gene ontologies, for example [56–58] and generate hypotheses about genes and traits that contribute to environmental adaptation in this system [36,38]. For example, in this study, while many candidate genes relate to phenotypes known to vary with latitude, such as body size, the results of functional analyses suggest additional phenotypes to consider, such as those relating to immunity, circadian rhythm, temperature sensing, and cardiac function.

Nevertheless, many of the traits of interest in this system are complex (e.g., body size and aspects of metabolism), where effects of individual variants are expected to be small. Moreover, most candidate genes did not contain candidate SNPs that were amino-acid changing, suggesting much of the response to selection is due to changes in gene regulation. To try to better understand how candidate genes affect phenotypes, studies of wild house mice in the Americas have been integrative [36,38,40,61,65,72], including phenotyping in the wild and in the lab, expression studies in wild and laboratory populations, and GWAS, in addition to selection scans. For example, lab-born descendants of mice from cold environments are larger than lab-born descendants of mice from warm environments [38,65,72] and GWAS across the Americas for body weight identified eight candidate genes. All but one of these genes was also identified in selection scans, and two of them were identified in all three transects: *Snx25*, which is linked to activity, cardiac function, and more recently, circadian pace-making [68] and *Ralgapa2*, which is linked to neoplasm and glucose homeostasis [67]. The significant overlap between the selection scan and GWAS results highlights these genes as promising candidates for further study.

We can also bring together the candidate genes identified here in all three transects with published functional data and gene expression studies [36,40,61,65]. The use of replicated transects provides strong evidence that a core set of genes with diverse functions contribute to environmental adaptation in the Americas. For example, *Mlh1* was a candidate in all three transects and a top candidate in two. Mutants in *Mlh1* affect aspects of immune system function among other phenotypes. *Mlh1* differs in expression both among inbred strains from the Americas [61,65] and among wild mice from opposite ends of the ENA transect [40], and it was associated with a *cis*-eQTL in ENA. Another example comes from the *Trpm* gene family. TRPM channels act as cellular sensors with impacts on diverse physiological processes, including temperature sensing, mineral homeostasis, cardiac rhythm, and immunity [73]. Multiple genes in the *Trpm* family have been linked to response to environmental factors like temperature and light, among others [74–77]. *Trpm2* was identified as a candidate gene for latitude and MAT in all transects [36,38] and was linked to differences in gene expression in strains derived from different populations of the Americas [36]. Mutant phenotypes in *Trpm2* relate to immunity [78] and experimental results link *Trpm2* to sensitivity to warmth [76] and insulin secretion [79]. *Trpm6* was identified as a candidate in all three transects, functions in Mg+ transport, and was linked to differential expression under different temperature regimes in lab strains derived from New York and Brazil [61]. While these results do not provide definitive links between genes in the *Trpm* family and specific phenotypes, they do identify a potential role for genes that mediate temperature sensing in adaptation to novel climates.

### Shared responses to environmental variation

Clinal or ecotypic approaches have long been used to detect selection in wild populations [2,80–84]. Clinal variation in traits like allozyme polymorphisms [80,81] and diapause [84] across environmental gradients have been interpreted as signals of potential adaptive significance. Shared responses to selection and specifically parallel patterns of clinal or ecotypic variation have been of particular interest, both because they provide strong evidence of adaptation and because they help us better understand to what extent evolution is predictable. Theory suggests a number of factors that might influence the predictability of evolutionary outcomes across parallel gradients or ecotypes including the mode of selection, similarity of trait optima, the amount of divergence time, factors related to the architecture of the trait (e.g., the number of loci underlying variation in the trait, pleiotropy, genetic redundancy), and population specific factors (e.g., initial allele frequencies, mutation rates, gene flow) [29]. Moreover, in many systems, selection is expected to be multivariate, with multiple factors acting on traits in potentially complex ways, resulting in scenarios in which clinal trait variation can occur even when underlying allele frequencies are not clinal [54].

With data from three transects across two continents, we can address how much of the response to selection across latitudinal gradients is shared and what that can tell us about adaptation in this system. Two major findings emerge. First, changes in putatively regulatory regions dominate signals of selection in all three transects. While the relative contribution of regulatory and amino acid changing mutations to differences in phenotype is not known, the overwhelming and consistent signal suggests a key role for regulatory changes in rapid adaptation to climate across latitude. This result is similar to what has been found in another well-known example of parallel adaptation, the three-spined stickleback, in which freshwater habitats have been repeatedly and independently invaded from marine source populations [19].

Second, while most candidate genes are unique to individual transects, there is evidence for shared responses to selection in this system as well as some evidence for parallel clines. Overlap among candidate genes from the three transects was significantly more than expected by chance for both latitude and mean annual temperature, with ~16% of candidates shared between any two transects on average and ~7% of candidates shared among all three transects. Moreover, the direction of allele frequency shifts in overlapping candidate genes was shared in all three transects significantly more often than expected by chance. Adaptation across latitudinal gradients in house mice undoubtedly encompasses changes in many different traits, many of which are likely complex, such as body size, aspects of metabolism, immunity, and behavior. Shared responses to selection are expected to be more common for simple traits in which the mutational targets are small compared to complex, highly polygenic traits [85]. However, in a meta-analysis of published studies across a range of taxa, Conte *et al*. (2012) showed that the probability of parallelism increases with decreasing age of the common ancestor of the compared taxa [86]. Broadly, the results of this study are consistent with theoretical predictions. In this case, the traits of interest are largely complex, and we have observed mostly unique signals of adaptation. Nevertheless, there is significant evidence of a shared response to selection; house mice in the Americas are of very recent origin, and responses to selection in the three transects are almost certainly fueled by standing genetic variation from European populations.

There are now many diverse empirical examples of independent, shared responses to selection in response to environmental variation within species. As mentioned above, sticklebacks are perhaps the most well-known example in vertebrates with extensive genomic data [11,19,87]. While EDA stands out as a shared locus of major effect that contributes to adaptation in the marine/freshwater transition [87], data suggest extensive common responses across the genome arising from selection on standing variation [11,19]. Comparisons among lakes and streams also suggest that unique responses might relate to population specific environmental factors [88] and data from throughout the range of the species suggest that shared genetic responses may be common in the Eastern Pacific because of a unique demographic history [89]. In a genomic analysis of four global regions of marine to freshwater transitions [8] most signals of selection associated with environmental and phenotypic variation were unique to individual regions, but there was significant and compelling evidence of overlapping candidate regions. While there are clear differences, the genome-wide nature of response to selection and the strong signal of regulatory change in sticklebacks mirror to some degree the results of this study, perhaps in part because in both cases, putatively adaptive phenotypes have a complex genetic basis, adaptation is relatively recent, and standing variation is expected to be the foundation of such adaptation.

As in this study, studies of selection in *Drosophila melanogaster* have spanned latitudinal clines across continents [reviewed in 13]. Populations along the east coast of North America, which experiences significant, correlated, and predictable shifts in climatic variables with latitude, have been extensively studied for decades [81,84,90]. Populations in eastern Australia have also been extensively studied [10,91–93] over a latitudinal gradient with similar shifts in climatic variables facilitating studies of shared responses to selection and parallel adaptation across the two continents [12,94–97]. *D*. *melanogaster* is cosmopolitan and there are also many comparative global studies of adaptation [94,98,99]. While many of these studies have focused on specific phenotypes or candidate genes/alleles [12,94–99], some studies have used a genome-wide approach to investigate environmental adaptation in specific transects [100–102] as well as to investigate parallel adaptation [15]. Moreover, recent genome wide approaches to parallelism have expanded to include Europe [103] and to facilitate worldwide comparisons [104].

Studies in *D*. *melanogaster* (both candidate gene and genomic) have, in some cases, found significant evidence of shared response to selection over latitudinal gradients. Perhaps most well-known, the “fast” allele of ADH is consistently at higher frequency in higher latitudes, providing evidence of parallel adaptation at this locus [81,98,99]. On the other hand, some examples underscore the biological complexities that can underly shared responses to selection. *Couch potato* (*cpo*) stands out as an example for which the connection to function (diapause) and fitness has been directly demonstrated [84]. There are latitudinal clines in allele frequencies in eastern North America consistent with those impacts on function and fitness [84]. Clinal patterns from eastern Australia are broadly similar [105], but the details of the clines vary in many ways, including the nature of the cline and associated phenotypes [105,106; reviewed in 13]. Moreover, parallelism on both continents is complicated by demographic history, namely colonization from both Europe and Africa [e.g., 107].

Genomic studies have found extensive evidence of clinal variation in both North America and Australia [101,102] and the identified candidate genes are diverse, suggesting that many different traits may be involved in adaptation. Similar to our results with mice in the Americas, differentiation in flies was especially high in regulatory regions in North America [102]. Comparing the results of two studies [101,102], Adrion *et al*. (2015), noted that ~31% of the genes that are differentiated between populations at the ends of the cline in North America show the same pattern in Australia [13]. Reinhardt *et al*. (2014) found substantial evidence of parallel adaptation in genomic analysis of clines in both North America and Australia [15]. While an inversion contributes to that pattern, there is also evidence of parallelism throughout the genome. The authors point out that the large number of parallel candidates along with the range in the magnitude of differentiation of these candidates suggest many targets of selection. While the degree of overlap in *D*. *melanogaster* is higher than seen in this study, the greater number of unique candidates and the diversity of candidates throughout the genome in both systems is consistent with selection on complex traits.

## Conclusion

House mice arrived in the Americas in association with human colonization, quickly and successfully establishing populations in a variety of climates and habitats. Consistent with their natural history, genetic differentiation among populations is relatively high and, while evidence for isolation by distance is restricted to South America, populations across the Americas cluster by continent and by region within continent. Bringing together results from three independent transects, we demonstrate that adaptation across latitudinal gradients in the Americas is largely driven by unique changes in putatively regulatory regions. However, significant overlap of candidate genes among transects and the generally consistent direction of allele frequency shifts in shared candidate genes provides evidence for a shared response to selection and parallel adaptation. The wealth of functional data available for house mice together with gene expression studies in wild populations and new wild-derived inbred strains from the Americas help connect these candidate genes to traits with the potential to affect fitness. While much more is now known about the genetics of wild house mice in the Americas, population genomic data at this geographic scale combined with the functional resources generated (new wild-derived strains, phenotype data, and gene expression data) point to great potential for continued investigation of the genetic basis of adaptation in this system and more broadly, to the connection between genotype and phenotype in house mice [72].

## Materials and methods

### Ethics statement

Animals were collected and sacrificed following protocols approved by the Institutional Animal Care and Use Committee (IACUC) of the University of Arizona and the Animal Care and Use Committee (ACUC) of the University of California, Berkeley. All wild-caught animals were collected with permits issued from Mexico, Brazil and Argentina.

### Sampling

Mice were collected using live Sherman traps from sites within eight sampling locations along a latitudinal transect in South America and from two sampling locations in Mexico (Fig 1A and S1 Table). When possible, mice were collected from ten or more sites within each sampling location and sites were at least 500 m apart to avoid the inclusion of close relatives. In some sampling locations, this collection scheme was not tractable and either fewer sites were included (Ushuaia, Argentina; Chiapas, Mexico) or some sites were less than 500 m apart (S1 Table). Sex and body size data were recorded for each mouse along with latitude, longitude, and elevation (S1 Table). Measures of size included total length, tail length, hindfoot length, and ear length as measured with a ruler and total weight (grams) measured using a micro-line spring scale. Animals were sacrificed in accordance with a protocol approved by the Institutional Animal Care and Use Committee (IACUC) of the University of California, Berkeley. Tissues including the liver, kidneys, and spleen were collected and either stored in liquid nitrogen or dry ice until transfer to a -80°C freezer or immersed in 96% EtOH that was drained and replaced after 24 hrs. and then stored at 4°C. Skins, skulls, and skeletons were deposited in the Museum of Vertebrate Zoology, University of California, Berkeley (S1 Table).

### DNA Extraction, library preparation, and sequencing

DNA was extracted and exome capture libraries were prepared as in Phifer-Rixey et al. (2018) [36]. Individuals were pooled for capture and each pool of enriched capture libraries was then sequenced on each of five lanes of an Illumina HiSeq4000 (150-bp paired-end) resulting in an average of approximately 6.4 GB of raw data per individual (S1 Table). Sequence data from three individuals (FMM273, FMM 275, FMM276, Rodonia, Brazil) was generated separately but with a similar capture, pooling, and sequencing approach.

### Exome-capture pipeline

The exome sequence data were cleaned, and adapters were removed with the program AdapterRemoval [108] using a minimum quality of PHRED ≥ 30. Then, we used the *Escherichia coli* genome (ASM584v2) to filter potential contamination. We mapped the exome raw reads against the *E*. *coli* genome, and we retained the unmapped reads using HISAT2 [109]. After cleaning and filtering, we retained more than 99% of the reads (S1 Table). On average, the sequence depth coverage per site was 33.6x and 92% of the targeted exome was covered. The resulting reads were mapped to the house mouse reference genome (GRCm38.p6) using BWA-MEM [110,111]. Aligned reads were sorted, duplicates were marked and removed, and the reads that aligned to chromosomes X and Y were extracted using Picard and Samtools software (https://broadinstitute.github.io/picard/) [112]. We followed the GATK Best Practices pipeline to identify artifacts or technical errors made by the sequencing machine using base calibrator tools (BQSR and ApplyBQSR). Additionally, we performed a local realignment for indels and a variant filtering using the variant quality score recalibrations (VQSRs [113]).

Based on the Bayesian method implemented in ANGSD [114,115], we estimated the allele frequencies and called SNPs for the six populations using the recalibrated bam files generated with GATK (calling genotypes with a posterior probability ≥ 95% and a *p-value* ≤ 10^3^; for additional details, see https://github.com/YocelynG/HouseMouse_EnvAdapt). Finally, we retained those variants that were present in at least 80% of the samples, obtaining a total of 271,720 SNPs.

### *Mus sp*. Genomic data and admixture analysis

To investigate genetic admixture between South American and North American populations and the historical relationships among *Mus musculus*, we included the previously published genomic information for 50 individuals of *M*. *m*. *domesticus* from Eastern North America [36], 50 individuals from Western North America [38], 10 *M*. *m*. *domesticus* from France and Germany, three *M*. *m*. *musculus* individuals each from the Czech Republic and Kazakhastan, and 10 individuals of *M*. *m*. *castaneus* [52] (S2 Table). All of these data are publicly available and were downloaded from the NCBI Sequence Read Achieve or the European Nucleotide Archive repositories (S2 Table).

We cleaned and filtered the raw reads using the same method applied to the data generated for this study. The resulting reads were mapped to the house mouse reference genome (GRCm38.p6) using BWA-MEM. The mapped reads were sorted, duplicates were marked and removed using picard and samtools. We used GATK pipeline to identify artifacts or technical errors (BQSR and ApplyBQSR). Additionally, we performed a local realignment for indels and a variant filtering using VQSRs [113]. Finally, from the genomic bam files, we extracted the exome coordinate regions using samtools, bedtools and bash scripts [116,117]. We used the autosomal recalibrated bam files and the software ANGSD [114] to calculate genotype likelihoods for polymorphic sites (for additional details, see https://github.com/YocelynG/HouseMouse_EnvAdapt).

To obtain an accurate estimate of the admixture proportions and the best genetic cluster value (K), we ran *NGSadmix* for several K values: K = 2 to K = 5, with 5,000 as the maximum number of EM iterations. We used the log-likelihood estimated for each K to calculate the Cluster Markov Packager Across K from Evanno using R scripts.

### Phylogenetic reconstruction

To investigate the phylogenetic relationships between the South American house mice populations and their close relatives from North America, we used the exome sequence data generated for this study, 86 individuals from Mexico and South America, and the exome data from the 100 individuals from North America included in the admixture analysis [36,38]. We also incorporated the genomic data of *M*. *spretus* (one individual, Project: PRJEB11742, sample: ERR1124353) (S2 Table) as an outgroup.

We used ngsDist (from ngsTools) [118] to estimate pairwise genetic distances, using the genotype likelihoods calculated for *M*. *m*. *domesticus* and *M*. *spretus* as an outgroup (with 100 repetitions of bootstrapping for node support). We used RAxML [53] to place support in the main tree, and FigTree software [119] to visualize the tree.

### Genetic differentiation between populations

To explore population structure in the house mouse populations from North America, Mexico and South America, we used ngsCovar software [118] to generate a genetic covariance matrix from the genotype posterior probabilities) generated from the autosomal bam files of 186 individuals. We included sites with a minimum MAF (—minMAF parameter) ≥ 5%, and a *p-value* ≤ 10^3^ (for additional details, see https://github.com/YocelynG/HouseMouse_EnvAdapt). We used R to perform the eigenvalue decomposition and generated the PCA plot using the three principal components. With ANGSD, we estimated *F*_*st*_ for each pair of populations (North and South America), using the unfolded pairwise site frequency spectra (SFS) as priors for the allele frequency probabilities at each site. Additionally, we used VCFtools to calculate pairwise Weir and Cockerman’s *F*_*st*_ for all the populations. Finally, for the populations from South America, we analyzed the relationship between genetic distance and the geographic distance by performing a Mantel Test using the R packages vegan, adegenet and hierfstat [120–122]

### Relatedness analysis

To infer relatedness between pairs of individuals in the South American populations, we used ANGSD to estimate the genotype likelihoods and allele frequencies (including sites with a minimum MAF ≥ 5%, and a *p-value* ≤ 10)^3^ and the program ngsRelate [123] to calculate different relatedness and inbreeding coefficients. To identify close relatives, we used the relatedness coefficient proposed by Hedrick and Lacy [124]. Those pairs of individuals with a relatedness measure above 0.25 (equivalent to half-siblings) were considered relatives resulting in the removal of 14 individuals from Mexico and South America (S1 Fig). Relatedness heatmaps were generated using R (R Team Core).

### Environmental association analysis

We used the Arctos database to obtain the geographic coordinates of each locality (latitude and longitude) for the 186 individuals of house mice from South America, eastern and western of North America (S1 Table). Based on the geographic coordinates, we extracted the 19 bioclimatic variables from WorldClim database [125] with 30 seconds spatial resolution for each individual, using the package raster in R [126]. We performed a PCA using the 19 bioclim variables to explore the climatic variability of our samples and to identify the most informative environmental variables (S5 Table). Moreover, we tested the relationship between latitude and the two bioclim variables (Bio 1- Annual Mean Temperature and Bio 14 –Precipitation of the Driest Month) calculating Pearson’s correlations.

To identify candidate genes underlying environmental adaptation, we performed a population genomic scan for selection using LFMM [4] and three environmental variables (Latitude, Bio 1, and Bio14). LFMM used a Bayesian bootstrap algorithm that accounts for population structure while identifying genetic polymorphisms that exhibit correlations with variables of interest (environmental measures or phenotypic traits). For each environmental variable, we ran LFMM (25 repetitions, burn-in = 100,000 and 500,000 iterations), using K = 3 for South America, and a K = 2 for the eastern and western transect. For each LFMM result, we estimated the genomic inflation factor (λ), and the *p-values* were adjusted to control for the false discovery rate. We identified candidate SNPs using a threshold *q-value* ≤ 0.05 and |*z-score|* ≥ 2.

To explore the potential functional significance of the candidate SNPs identified, we used Ensembl’s Variant Effect Predictor command line software [127] with the house mouse reference genome (GRCm38.p6). Many SNPs had more than one potential functional consequence. To classify the SNPs annotated based on their “primary” functional consequence we followed the scheme proposed by Phifer-Rixey et al. (2018) [36]: 1) missense, stop lost or stop gain; 2) 3’ or 5’ UTR; 3) synonymous; 4) non-coding exon variants or non-coding transcript variants; 5) intron or splice site variants; 6) downstream or upstream variants.

Additionally, we extracted from The Mouse Genome Informatics Database (MGI) [56–58] the gene ontology terms (GO) [128] associated with the genes annotated in each LFMM analysis. We conducted an enrichment analysis using GOwinda [55] which accounts for potential biases introduced by variation in gene length. First, using all candidate SNPs, we ran the analysis in *snp* mode and then in *gene* mode. Both of these modes make simplifying assumptions. The *snp* mode assumes that all SNPs are independent, including those within the same gene. In contrast, *gene* mode assumes that all SNPs within a gene are completely linked. Therefore, *snp* mode likely results in an overestimate of gene enrichment when there is linkage disequilibrium within genes and *gene* mode will result in an underestimate when SNPs within genes are in linkage equilibrium [55]. The average distance over which LD decays in mouse populations is typically somewhere between these two extremes which suggest that both assumptions are likely to be violated [129]. Therefore, we also implemented a third approach, choosing *snp* mode but using SNPs that had been pruned based on LD using PLINK2 in both the target and background set [130]. Pruning was based on an r^2^ of 0.5 with non-overlapping 50 kb windows. For *gene* and *snp* mode, we used all called SNPs as a background set and conducted the analysis using the exon parameter, associating SNPs within exons with genes. We performed 10,000 simulations and the GO enrichment *p-values* were corrected using a False Discovery Rate approach.

We used the R package “VennDiagram” [131] to identify those genes shared among LFMM environmental variables results, and also among transects. With the R package “RegionR” [132], we performed permutation analysis to test if the genes shared between transects (South America–ENA, South America–WNA, ENA–WNA) were significantly greater than expected by chance. First, we generated a data frame using the genomic regions of those SNPs identified as a candidate for each environmental variable for each transect, using the function *toGRanges*. To evaluate if the overlap between the candidate genes shared among two transects is higher or lower than expected, we used the function *overlapPermTest*. We performed three independent permutation tests for each environmental variable, evaluating the overlap between 1) South America–ENA; 2) South America–WNA; and 3) ENA–WNA, running 100,000 replicates with replacement, calculating the *p-value* and *z-score*.

To investigate the direction of allele frequency changes, we generated VCF files separately for each transect, incorporating population data from Edmonton and Tucson (WNA), from New Hampshire–Vermont and Florida (ENA), and from Gaiman and Manaus (SA). We only included sites with data for at least 60% of the individuals in each population. We identified all shared SNPs in candidate genes shared among all three transects. Then, we characterized the direction of the allele frequency shift across the relevant gradient (latitude, AMT, or PDM) and determined whether the direction was the same in all three transects. To evaluate the significance of the results, we used χ^2^ tests with the expectation that 25% of genes should share directional shifts among all three transects by chance (S13 Table). In addition, we used permutation tests with 1,000 iterations, selecting with replacement the same number of genes as in the overlap (101, 116, or 5) from the full set of genes with shared SNPs to determine if the number of genes at which there was parallel change was more than expected by chance given genome wide patterns (https://github.com/YocelynG/HouseMouse_EnvAdapt). There were data for 16,984 genes, of which 2,243 showed parallel changes in allele frequency in all three transects. Given the results, we calculated the cut-off values corresponding to 5% for the distributions as well as the *z-score* and *p-value* for the observed number of genes with parallel changes for each environmental factor (S13 Table).

### Genome-wide association analysis for body weight

We conducted a genome wide association analysis to identify genes that contribute to variation in body weight using the program GEMMA [66] that implements an algorithm of linear mixed models. We used the body weight data from 116 adult house mice from the Americas. We excluded individuals reported as juveniles, subadults, pregnant, those with undetermined reproductive status, and those that were weighed after being housed in a laboratory colony. We used a total of 163,121 loci (filtered by minor allele frequency > 5%, and < 10% of missing data) to generate a genotype matrix as an input for GEMMA. We ran GEMMA using the body weight as a phenotype, controlling for relatedness among individuals and population structure, and using sex as a covariate to reduce the number of false positives. We adjusted *p-values* using a false discovery rate correction. Finally, the SNP candidates were annotated using the Ensemble’s variant predictor command line program. To test whether the overlap between GWAS candidates and the LFMM selection scans was more than expected by chance, we used a permutation test implemented in RegionR [132] as described above.

## Supporting information

S1 Table List of 86 wild-caught house mice individuals (*Mus musculus domesticus*) collected in Mexico (N = 10), Brazil (N = 60) and Argentina (N = 26). Table contains: Collector’s number, SRR ID, Museum of Vertebrate Zoology catalog number, exact collecting locality, latitude, longitude, sex, reproductive data, measurements of length and body weight, and data of exome sequencing (number of reads, length of reads and coverage).
(XLSX)

S2 Table Sample information for the European samples of *Mus musculus musculus*, *Mus musculus domesticus*, *Mus musculus castaneus*, and *Mus spretus* from Harr et al. (2016; Doi: 10.1038/sdata.2016.75) [52] included in our analyses. (XLSX)

S3 Table Pairwise differentiation (*F*_*st*_) across the three transects: South America, East and West of North America. (XLSX)

S4 Table Values for bioclimatic environmental variables for sampled populations of *M*. *musculus domesticus* across the Americas. (XLSX)

S5 Table Loadings of bioclimatic variables for the first five principal components for the analysis of climate data for all included populations in the Americas. (XLSX)

S6 Table Results of Latent Factor Mixed Model (LFMM) analysis for the each of three variables (latitude, MAT, PDM) in South America populations as well as information about top candidates and shared candidates, and the allele frequencies. (XLSX)

S7 Table The distribution of candidate SNPs identified in LFMM analyses of South American populations and all SNPs included in the analyses across predicted functional consequence categories. (XLSX)

S8 Table The results of enrichment and functional analyses for candidates identified using LFMM of South American populations. (XLSX)

S9 Table Classification and annotation of SNPs identified as candidates in LFMM analyses of North American populations with LAT, MAT, and PDM. (XLSX)

S10 Table Proportion of genes shared between and across all transects for each variable. (XLSX)

S11 Table Pairwise permutation test results for overlap among candidate genes for each environmental variable identified across the three transects, South America (SA), Eastern of North America (ENA), and Western of North America (WNA), using 10,000 permutations. The number of genes shared for each variable are described in Fig 4.
(XLSX)

S12 Table Functional information for candidate genes shared across the three transects for each environmental variable. (XLSX)

S13 Table Results of analyses of the direction of allele frequency changes at shared SNPs in candidate genes for the populations at the ends of each of the three transects. (XLSX)

S14 Table Gene annotations and functional information for candidate SNPs identified via GEMMA for body weight. (XLSX)

S1 Fig Heatmap of pairwise relatedness coefficients between individuals from the same population using the relatedness estimator R_AB_, described by Hedrick and Lacy (2014) [124]. Individuals that were removed because they were close relatives to another sampled mouse (with a pairwise relatedness value greater than 0.25) are shown in bold.
(TIF)

S1 Text Alternative language abstract (Spanish and Portuguese) and Alternative language author summary (Spanish and Portuguese). (DOCX)
