# Supplementary figures and images for: Across two continents: The genomic basis of environmental adaptation in house mice (Mus musculus domesticus) from the Americas
Source: PLoS Genet. 2024 Jul 5;20(7):e1011036. doi: 10.1371/journal.pgen.1011036 (PMC11253941; doi:10.1371/journal.pgen.1011036)

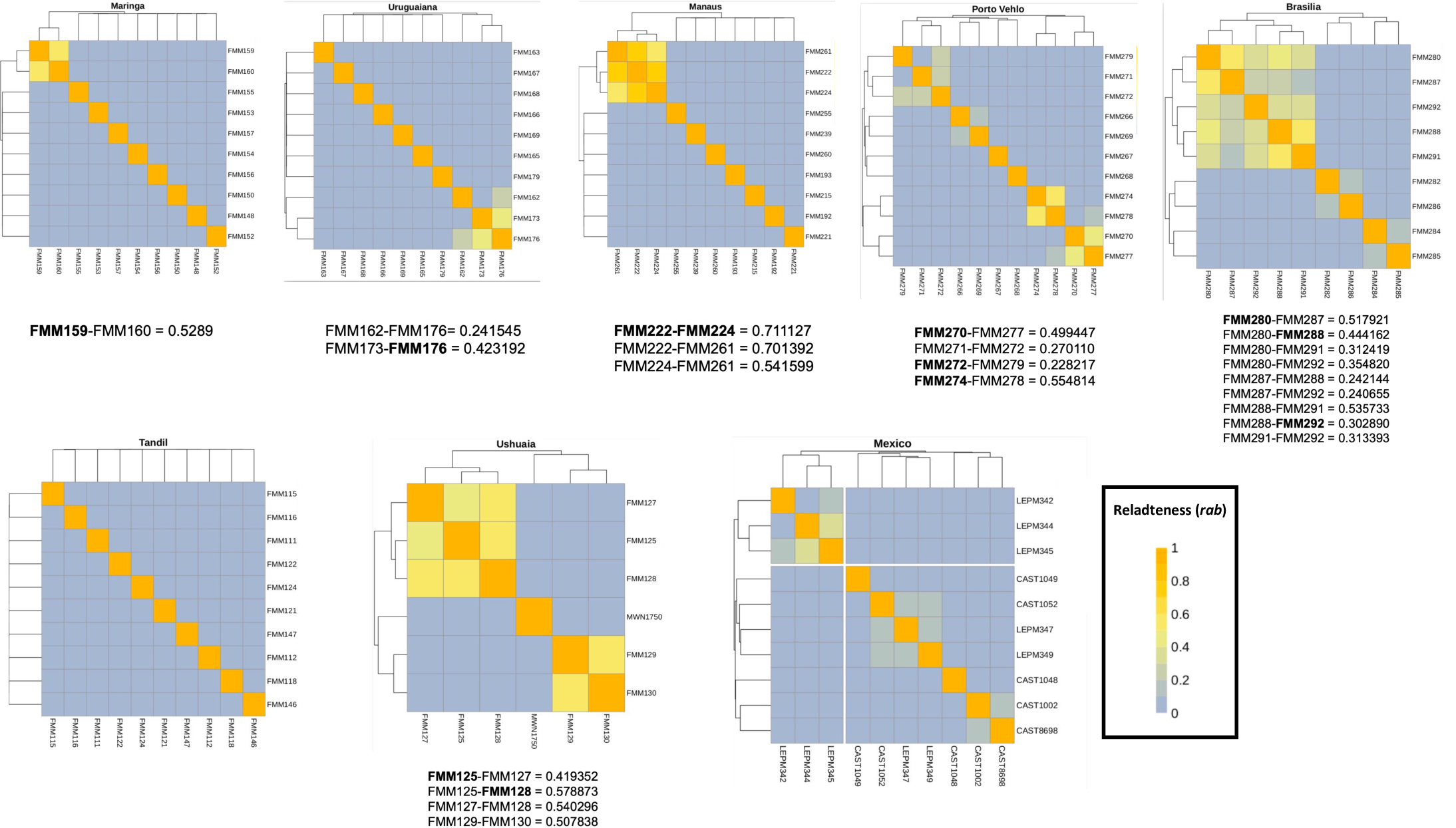

Supplement: S1 Fig — Individuals that were removed because they were close relatives to another sampled mouse (with a pairwise relatedness value greater than 0.25) are shown in bold. (TIF) [file pgen.1011036.s015.tif]
