# Supplementary material for: Across two continents: The genomic basis of environmental adaptation in house mice (Mus musculus domesticus) from the Americas
Source: PLoS Genet. 2024 Jul 5;20(7):e1011036. doi: 10.1371/journal.pgen.1011036 (PMC11253941; doi:10.1371/journal.pgen.1011036)
Supplement: S1 Text — (DOCX) [file pgen.1011036.s016.docx]

**Supporting Information**

**Supporting Information Index**

-Alternative language abstract [Resumen (Spanish), Resumo (Portuguese)]

-Alternative language author summary (Resumen del autor (Spanish) Resumo do autor (Portuguese)]

**Alternative language abstract**

Resumen

La repetibilidad de clinas a lo largo de gradientes ambientales representa una fuente importante de procesos de adaptación. El ratón doméstico (*Mus musculus domesticus*), es una especie que fue introducida a las Américas por los colonizadores europeos y que actualmente se encuentra distribuida desde Tierra del Fuego hasta Alaska. Una multiplicidad de elementos del clima, como la temperatura, presentan una variación predictible según la latitud en el continente americano.

Previos estudios realizados en poblaciones de ratones de Norteamérica a lo largo de gradientes latitudinales han identificado evidencia de adaptación al ambiente en rasgos asociados al tamaño corporal, metabolismo y comportamiento, además se han identificado genes candidatos por medio del escaneo del genoma. En este trabajo, investigamos las señales genómicas de adaptación ambiental en Sudamérica, y los procesos de adaptación paralela a lo largo de múltiples transectos latitudinales en las Américas. Primero, se hizo una búsqueda a lo largo del genoma de aquellos loci que presentaran señales de selección asociados con variación climática en poblaciones de ratones distribuidas a lo largo de un transecto latitudinal in Sudamérica, corrigiendo por la estructura poblacional. Consistente con previos resultados, la mayoría de los SNPs identificados tuvieron una función regulatoria. Los genes que contienen SNPs con mayor significancia, tienen funciones asociadas con rasgos en peso y tamaño corporal, metabolismo, inmunidad, grasas, desarrollo y funcionamiento de los ojos, del sistema cardiovascular y renal. Posteriormente, comparamos nuestros hallazgos con los resultados previamente publicados de dos transectos latitudinales en Norteamérica. Encontramos que la mayoría de los genes candidatos obtenidos son únicos a cada transecto. Asimismo, identificamos un sobrelape de genes candidatos que fueron obtenidos independientemente en los tres transectos analizados, con funciones diversas, asociadas con metabolismo, inmunidad, función del sistema cardiaco, ritmo circadiano, entre otras. También, se observaron cambios paralelos en las frecuencias alélicas en genes candidatos a lo largo de gradientes ambientales. Por último, utilizando los datos de los tres transectos, se identificaron genes asociados con la variación en peso corporal. Estos hallazgos proporcionan evidencia de procesos de adaptación paralela, así como la identificación de genes asociados con adaptación ambiental en poblaciones del ratón doméstico en América del Norte y América del Sur.

Resumo

A repetibilidade dos clines ao longo de gradientes ambientais representa uma importante fonte de processos adaptativos. O rato doméstico (*Mus musculus domesticus*) é uma espécie que foi introduzida nas Américas pelos colonizadores europeus e está atualmente distribuída do Terra do Fogo até o Alasca. Alguns elementos climáticos, como a temperatura, apresentam uma variação previsível de acordo com a latitude nas Américas. Estudos anteriores em populações de ratos da América do Norte ao longo de gradientes latitudinais identificaram indícios de adaptação ao ambiente em características associadas ao tamanho do corpo, ao metabolismo e ao comportamento, tendo sido identificados genes candidatos por meio da análise do genoma. Neste artigo, investigamos os sinais genômicos de adaptação ambiental na América do Sul e se existem processos de adaptação paralela ao longo de múltiplos transectos latitudinais nas Américas. Em primeiro lugar, realizamos uma busca em todo o genoma por identificar loci que apresentassem sinais de seleção associados à variação climática em populações de ratos distribuídas ao longo de um transecto latitudinal na América do Sul, considerando a estrutura populacional. Consistentes com resultados anteriores, a maioria dos SNPs identificados apresentava funções regulatórias. Os genes que contêm os SNPs com os valores mais significativos estão associados a características de peso e tamanho corporal, metabolismo, imunidade, gordura, desenvolvimento e função dos olhos, sistema cardiovascular e renal. Combinamos as nossas descobertas com resultados publicados anteriormente em dois transectos latitudinais na América do Norte. Constatamos que a maioria dos genes candidatos obtidos é exclusiva de cada transecto. Também, identificamos uma sobreposição de genes candidatos que foram independentes obtidos nos três transectos analisados, com funções diversas associadas ao metabolismo, imunidade, função do sistema cardíaco, ritmo circadiano, entre otras. Também, foram observadas alterações paralelas nas frequências aleatórias dos genes candidatos ao longo dos gradientes ambientais. Finalmente, utilizando os dados dos três transectos, foram identificados os genes associados à variação do peso corporal. Nossos resultados fornecem evidências de processos de adaptação paralela e a identificação de genes associados à adaptação ambiental em populações de rato doméstico na América do Norte e América do Sul.

**Alternative language author summary**

Resumen del Autor

Desde su llegada con las colonizaciones europeas, el ratón doméstico se ha distribuido exitosamente por las Américas. Estudios proveen evidencia de que las poblaciones de ratones de Norte América se han adaptado desde el inicio de la colonización, y a lo largo de gradientes latitudinales, han evolucionado paralelamente tanto fenotipos (por ejemplo, tamaño corporal y conductual), como un significante sobrelape de genes con señales de selección. En este trabajo, investigamos la genética detrás de la adaptación al ambiente en poblaciones de ratones de América del Sur. Identificamos que las poblaciones de América del Sur evolucionaron independientemente de las poblaciones de Norte América. Asimismo, identificamos genes candidatos a la adaptación al ambiente que están relacionados con rasgos en tamaño corporal, metabolismo, inmunidad, funciones del ojo, termorregulación y con el sistema cardiovascular. Se incorporaron los datos de los tres transectos a lo largo de ambos continentes para explorar si la adaptación ambiental puede ser predecible, con cambios genéticos que ocurren paralelamente en respuesta a condiciones ambientales similares. Encontramos que la mayor parte de las adaptaciones al ambiente no presentan cambios en la secuencia de aminoácido. Sin embargo, estos cambios se encuentran en regiones reguladoras. A pesar de que la mayoría de los genes candidatos son únicos a cada transecto, hay una significativa proporción compartida. Asimismo, observamos cambios paralelos en las frecuencias alélicas entre los genes candidatos compartidos, por ejemplo, cambios en la misma dirección a lo largo de diferentes gradientes latitudinales. Este grupo de genes candidatos que independientemente se encontraron en los tres transectos, probablemente desempañan un papel relevante en la adaptación al ambiente en las Américas. Por último, combinando los datos de los tres transectos se identificaron genes asociados con la variación del peso corporal. Estos resultados resaltan la significancia de estudiar poblaciones silvestres de esta especie que es un sistema modelo en la genética.

Resumo do autor

Desde a sua chegada com a colonização europeia, o rato doméstico tem sido distribuído com sucesso por todo as Américas. Estudos fornecem evidências de que as populações de ratos da América do Norte se adaptaram desde o início da colonização e, ao longo dos gradientes latitudinais, tanto os fenótipos (como tamanho do corpo e comportamentos) como os genes com sinais de seleção evoluíram em paralelo. Neste artigo, investigamos a genética por trás da adaptação ao ambiente em populações de ratos da América do Sul. Identificamos que as populações sul-americanas evoluíram independentemente das populações América do Norte. Também identificamos genes candidatos à adaptação ambiental relacionados a características como tamanho do corpo, metabolismo, imunidade, função ocular, termorregulação e sistema cardiovascular. Incorporámos dados dos três transectos em ambos os continentes para explorar se a adaptação ambiental pode ser previsível, com alterações genéticas que ocorrem em paralelo em resposta a condições ambientais semelhantes. Descobrimos que a maioria das adaptações ao ambiente não apresenta alterações na sequência de aminoácidos. No entanto, essas mudanças são encontradas em regiões reguladoras. Embora a maioria dos genes candidatos seja exclusiva de cada transecto, há uma proporção significativa que é partilhada. Também observámos alterações paralelas nas frequências de alelos entre os genes candidatos partilhados, ou seja, alterações na mesma direção ao longo de diferentes gradientes latitudinais. Este grupo de genes candidatos, que foram encontrados independentemente nos três transectos, provavelmente desempenham um papel relevante na adaptação ao ambiente nas Américas. Finalmente, ao combinar os dados dos três transectos, foram identificados os genes associados à variação do peso corporal. Estes resultados destacam a importância do estudo de populações selvagens desta espécie, que é um sistema modelo em genética.
